# Effects of age and circadian rhythm on vital parameters and erythrocyte osmotic fragility of donkeys during seasonal changes

**DOI:** 10.1371/journal.pone.0313780

**Published:** 2025-01-31

**Authors:** Moses Ayo-opemipo Olorunfemi, Abdulhakeem Binhambali, Victor Olusegun Sinkalu, Mohammed Babashani, Felix Uchenna Samuel, Joseph Olusegun Ayo

**Affiliations:** 1 Department of Veterinary Physiology, Faculty of Veterinary Medicine, Ahmadu Bello University, Zaria, Nigeria; 2 Department of Clinical Sciences, College of Veterinary Medicine, North Carolina State University, Raleigh, NC, United States of America; 3 Translation Research in Pain, College of Veterinary Medicine, NCSU, Raleigh, NC, United States of America; 4 Ahmadu Bello University Veterinary Teaching Hospital, Zaria, Nigeria; 5 National Animal Production Research Institute, Ahmad Bello University, Zaria, Nigeria; Universidade Federal de Mato Grosso do Sul, BRAZIL

## Abstract

This study investigates the effects of seasonal variations on the erythrocyte osmotic fragility and vital parameters of donkeys *Equus africanus asinus* at the National Animal Production Research Institute (NAPRI) in Shika, Kaduna State, Nigeria. The research focused on two key periods: the hot-dry season (April) and the rainy season (July). Twelve donkeys were classified into three age groups: young (1–3 years), adult (4–6 years), and old (7–9 years). Blood samples were collected at six-hour intervals over a 24-hour period, while dry-bulb and wet-bulb temperatures were measured to compute the temperature-humidity index (THI). Vital parameters including rectal temperature, respiratory rate, and heart rate were also recorded. Results showed higher dry-bulb temperatures (DBT) during the hot-dry season, with the lowest DBT of 12°C at 00:00 h and the highest of 25.5°C at 18:00 h. Young donkeys exhibited the highest erythrocyte osmotic fragility during the hot-dry season, while old donkeys showed elevated fragility during the rainy season. Results also demonstrated that erythrocyte osmotic fragility varied significantly with age and season, with young donkeys exhibiting the highest fragility during the hot-dry season at a 0.3% NaCl concentration. However, old donkeys showed increased fragility during the rainy season, which shows the influence of both age and environmental conditions on erythrocyte stability. Also, rectal temperatures were higher in young donkeys during the hot-dry season compared to adults, while heart rates showed significant elevation across all age groups during the rainy season. Overall, this study elucidates the physiological adaptations of donkeys to seasonal thermal stress, providing critical insights into their health management and welfare in varying climatic conditions. Understanding these dynamics is essential for optimizing donkey husbandry practices, especially in regions facing climate variability. These findings contribute valuable knowledge to the field of veterinary physiology and highlight the necessity of tailored management strategies to mitigate the impact of seasonal stressors on animal health.

## Introduction

**Fig 1:**
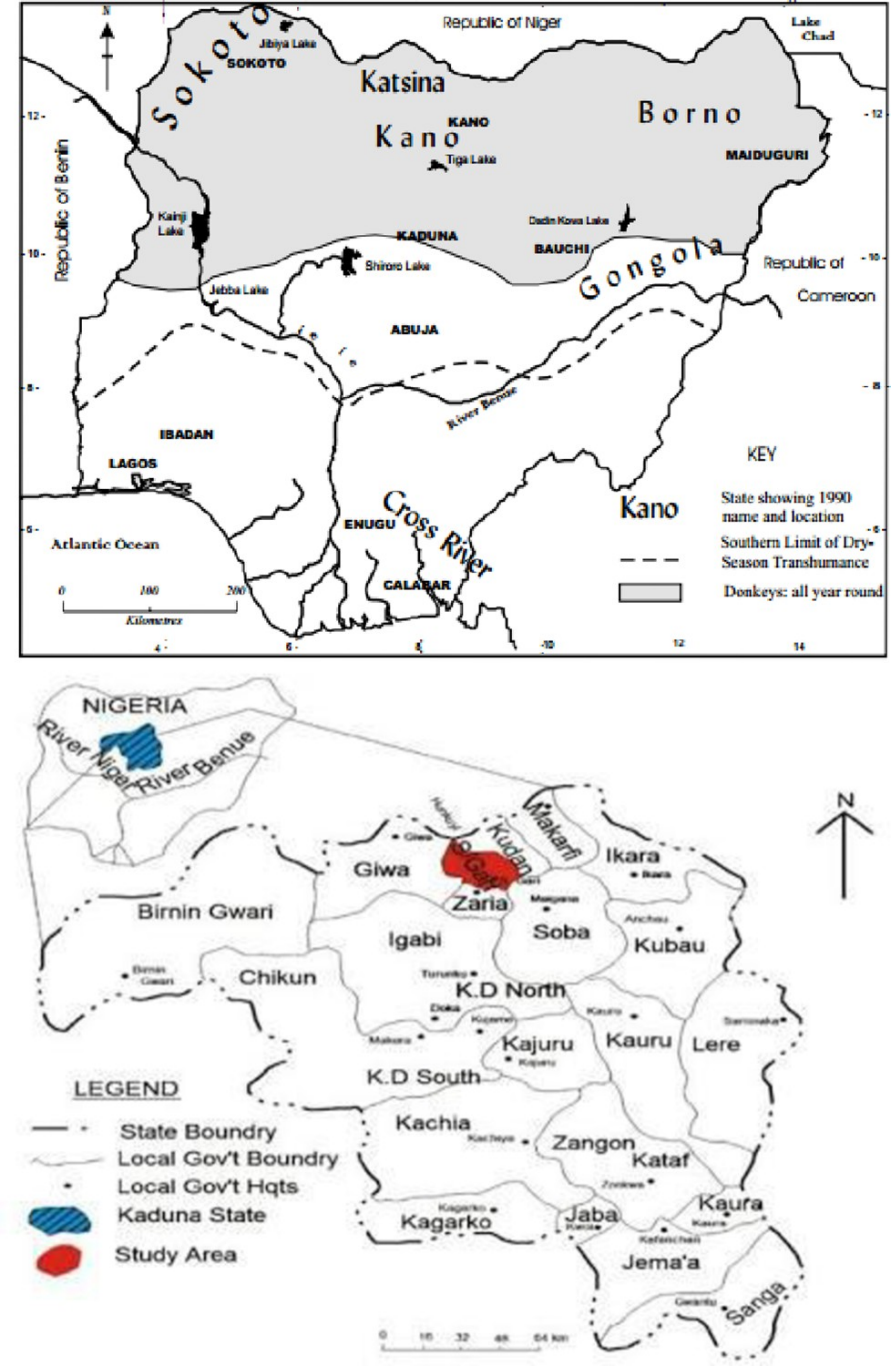
**a:** Distribution pattern of donkey in Nigeria (Image source: Google). **b:** A map showing Sabon Gari Local Government Area of Kaduna State, Zaria, Nigeria (Image source: Google).

The donkey, scientifically known as *Equus africanus asinus*, is a domesticated member of the Equidae family, with its wild ancestor being the African wild ass (*Equus africanus*). Found widely throughout Northern Nigeria, donkeys have long been integral to rural life, yet their utilization, distribution, and productivity under traditional management remain poorly understood [1]. However, there is a growing appreciation for their value due to their affordability, durability, and resistance to disease, particularly in rural households. Primarily employed as pack animals, donkeys are prevalent in regions influenced by Islamic culture and likely arrived in Nigeria through trans-Saharan trade routes, traversing the Nile, Sudan, and Chad, before reaching the Northeast via an East-West corridor [2]. While prevalent in the Northwest and North-central areas, their presence in the more humid southern states is limited due to susceptibility to internal parasites and trypanosomiasis. Despite these challenges, donkeys in Nigeria are known for their ability to work throughout the day with breaks for rest, water, and grazing. Training typically begins when they are 12–18 months old, with records indicating their use for plowing in various states such as Sokoto, Kaduna, Niger, Taraba, and Borno [3]. The distribution pattern of donkeys in Nigeria is depicted in [Fig 1].

Circadian rhythms, which encompass physical, mental, and behavioral changes following a daily cycle, play a crucial role in the lives of animals. These rhythms, primarily regulated by light and darkness, impact all vital parameters, with temperature being of particular interest. Vital parameters serve as crucial indicators of an animal’s health status and are instrumental in diagnosing diseases [4]. During clinical assessments, deviations from the normal range of vital parameters often serve as early signs of underlying health issues. The day-to-day fluctuations in the body temperatures of endothermic animals are not solely influenced by their physical and metabolic activities [5], but are also synchronized with daily changes in light intensity, temperature, and other environmental factors [6]. The ability of mammals to adapt to environmental changes is governed by a master circadian clock located in the suprachiasmatic nuclei of the anterior hypothalamus, acting as a conduit between the body and its surroundings, regulating oscillators in peripheral tissues [7]. Animal responses to environmental factors manifest through variations in cardio-respiratory responses, typically measured as fluctuations in heart rate and respiratory rate [8, 9]. These parameters, particularly heart rate and respiratory rate, are crucial in assessing donkeys’ responses to environmental changes, reflecting their adjustments in maintaining homeostasis [10]. Heart rate variation, for instance, mirrors the balance between sympathetic and parasympathetic tones, serving as an indicator of an animal’s stress response [9]. Despite the plethora of reviews on the biological rhythms of domestic animals’ body temperatures [11], there remains a scarcity of information regarding the influence of circadian rhythms on temperature and other vital parameters in donkeys [12]. Furthermore, there is a notable absence of literature describing the circadian rhythms of vital parameters specifically in donkeys.

Erythrocyte osmotic fragility tests measure red blood cell lysis under osmotic stress, serving as a valuable indicator of stress and oxidative stress indirectly in livestock [13]. Red blood cells, devoid of a nucleus and with a biconcave shape, carry oxygen to body tissues via hemoglobin molecules. Their susceptibility to oxidative stress is attributed to this oxygen delivery function and the concentration of polyunsaturated fatty acids in their cell membrane [14]. As animals age, the generation of reactive oxygen species increases, leading to adverse effects on red blood cells and variations in erythrocyte osmotic fragility [15–17]. Variations in rectal temperature, pulse rate, and respiratory rate in animals can be attributed to various factors, including age, exercise, excitement, environmental temperature, seasonal variation, pregnancy, and digestive tract fullness [18]. High environmental temperatures disrupt the thermoregulation system, adversely affecting the overall performance of donkeys and potentially leading to death [19]. Studies have shown altered vital parameters in packed donkeys, indicative of heat stress, which can be alleviated through antioxidant administration [20, 21]. However, donkeys may experience heat stress even without transportation or packing, especially considering factors such as age, time of day, and season. This issue poses a significant welfare concern for donkeys in countries like Nigeria. Research in physiology often employs erythrocyte osmotic fragility as an indicator of stress in packed or exercised donkeys, sometimes coupled with the administration of antioxidants. However, there is limited focus on heat stress in donkeys during their normal physiological state, highlighting the necessity for this study.

The overarching aim of this study is to assess the impact of age variation and circadian rhythm on vital parameters and erythrocyte osmotic fragility of donkeys during both the hot dry and rainy seasons in Nigeria.

## Materials and methodology

### Study location

**Fig 2:**
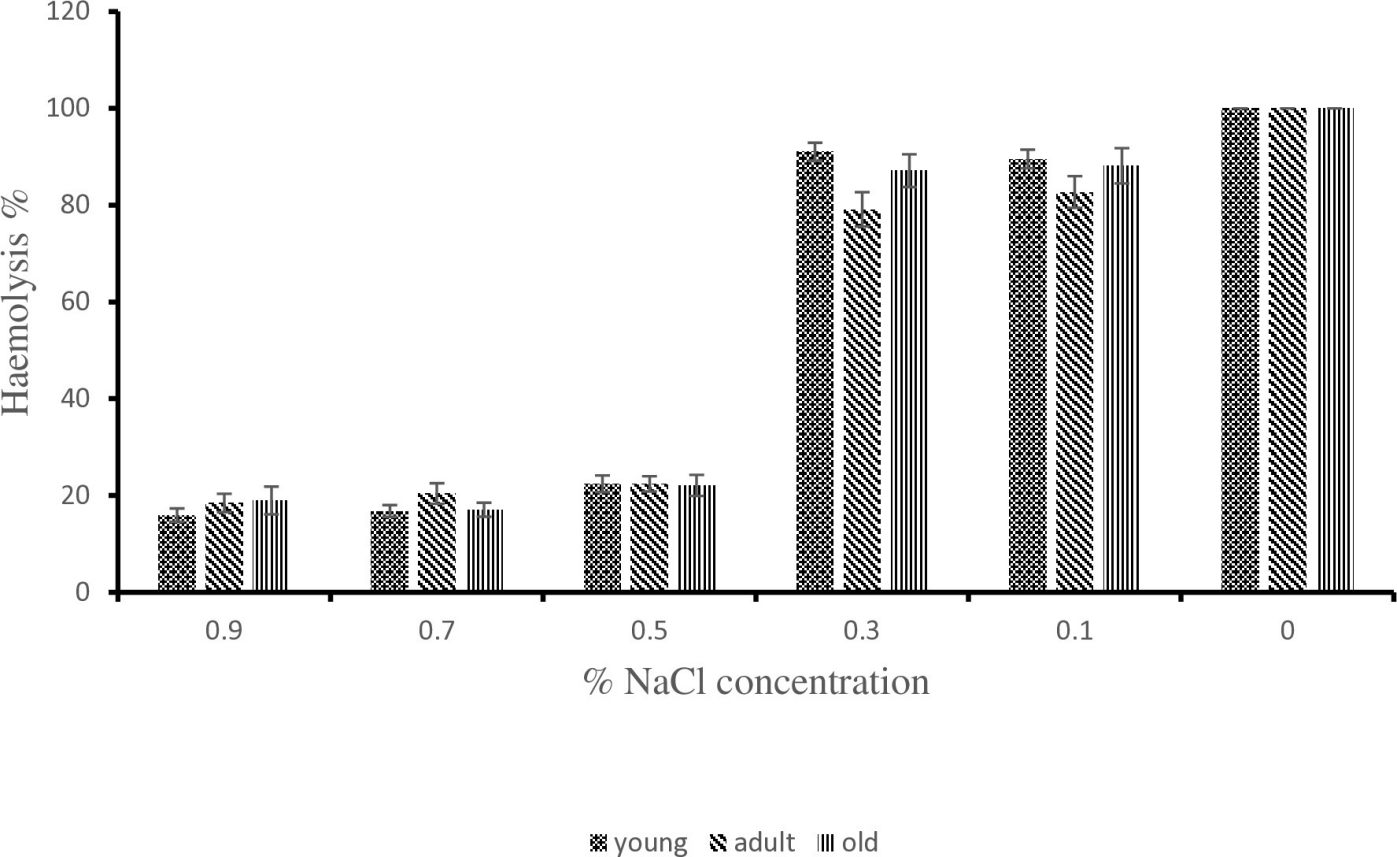
Effect of age on erythrocyte osmotic fragility of donkeys during the hot-dry season. ^a, b^: Means belonging to different age group and having different superscript letters are statistically significant (P < 0.05).

The research was carried out in the beef experimental unit belonging to the National Animal Production Research Institute (NAPRI), located in Shika [Fig 2] [22] between latitude 11°12’N and longitude 7°33’E, Kaduna state, located in the Northern Guinea Savannah zone of Nigeria and 610 meters above sea level. The climate is sub humid. Mean annual rainfall and temperature recorded at Samaru, 10km from Shika, were 1107 mm and 24.4°C respectively. The seasonal distribution of rainfall is approximately 0.1% in the late dry season (January—April), 25.8% in the early wet season (May—June), 69.6% in the late wet dry season (July—September) and 4.5% in the early dry season (October to December) [23].

### Seasons in Nigeria

Nigeria exhibits a tropical climate characterized by distinct rainy and dry seasons, the timing and intensity of which vary across the country’s regions. The climate diverges across three main zones: Southern Nigeria, characterized by an equatorial climate; Central Nigeria, experiencing a tropical climate; and Northern Nigeria, known for its arid conditions. Southern Nigeria encounters heavy rainfall throughout the year, with a relatively consistent temperature range and minimal fluctuations. In contrast, Central Nigeria witnesses well-defined wet and dry seasons, marked by heavy rainfall during the wet season and high temperatures during the dry season, accompanied by the West African trade winds originating from the Sahara desert to the north. Northern Nigeria, with its arid climate, receives minimal rainfall compared to other regions, and the rainy season is short-lived, lasting only a few months. The remainder of the year is typically characterized by dry and hot conditions. While temperature and humidity remain relatively stable throughout the year in Southern Nigeria, they exhibit significant variability in the north, particularly during the dry season, where the daily temperature range expands.

In summary, the duration of the rainy season diminishes from southern to northern Nigeria. In the North, the rainy season typically extends from mid-May to September, whereas in the south, it lasts from March to November, with a brief interruption known as the August break in August. The hot-dry season, also referred to as the long dry season, begins in late October in the south and lasts until early March, reaching peak dryness between early December and late February. In Northern Nigeria, the dry season spans from October to April [23].

### Experimental period

Blood samples were collected over two consecutive days, specifically on the 23rd to 24th of April, 2019, and the 3rd to 4th of July, 2019, representing the hot-dry and wet seasons, respectively, prevailing in the Northern Guinea Savannah zone of Nigeria.

### Experimental design

The donkeys were categorized into three groups based on their age, with each group consisting of four donkeys. Group I comprised donkeys aged between one to three years, classified as "Young." Group II encompassed donkeys aged four to six years, categorized as "Adult." Group III consisted of donkeys aged seven to nine years, designated as "Old.".

#### Thermal environmental parameters

Dry-bulb temperatures (DBT) and wet-bulb temperatures (WBT) were documented every 6 hours over a 24-hour period using a wet- and dry-bulb thermometer (Mason’s type, Zeal, England). Relative humidity was determined employing an Omsons hydrometric table (Narindra Scientific Industries, Haryana, India). The temperature-humidity index (THI) for each observation period was computed following the method described by Yousef [24], employing the following equation:

$$THI=(0.35\;t_{d}+0.65\;t_{wb})\;x\;1.8+32$$

Where t_d_ was the dry-bulb temperature (°C) and t_wd_ was the wet-bulb temperature (°C).

#### Vital parameters

Measurements of rectal temperature, heart rate, and respiratory rate were conducted at 6-hour intervals over a 24-hour period, commencing at 12:00 h on day 1 and concluding at 06:00 h (GMT +1) on day 2. Each donkey was gently restrained during the assessment process. Rectal temperature, serving as an indicator of body temperature, was measured using a digital thermometer (COCET, Kangfu Zhejiang Yuequing, China). The thermometer was inserted approximately 3.5 cm into the rectum via the anus until a beeping (alarm) sound signaled the completion of the recording. Respiratory rate was determined by observing and tallying the number of respiratory flank movements for one minute. Heart rate was assessed by auscultating the heart with a stethoscope (Sprague, Rappaport Type Stethoscope, England) positioned between the fourth and fifth ribs on the left side of each donkey, and the number of heartbeats per minute was recorded.

#### Blood sample collection

Blood samples were collected from each donkey at 6-hour intervals over a 24-hour period, starting at 12:00 h on day 1 and concluding at 06:00 h (GMT +1) on day 2. One milliliter of blood was drawn from the jugular vein of each donkey and transferred into separate EDTA bottles. Subsequently, the blood samples were transported to the Veterinary Physiology Laboratory, ABU, Zaria, for erythrocyte osmotic fragility tests.

### Laboratory investigation

#### Erythrocyte osmotic fragility test

Erythrocyte osmotic fragility was assessed following the procedure outlined by Oyewale et al. (2011) [25]. Briefly, 0.02 ml of blood obtained from each donkey in the respective age groups was added to labeled tubes containing increasing concentrations (0, 0.1, 0.3, 0.5, 0.7, and 0.9%) of phosphate-buffered sodium chloride (NaCl) solution at a pH of 7.4. After gentle mixing, the tubes were incubated at room temperature (25°C) for 30 minutes. Subsequently, the tube contents were mixed again and centrifuged at 400 g for 10 minutes. The optical density of the supernatant from each tube was measured spectrophotometrically at 540 nm using an SM 22 PC Spectrophotometer (Surgienfield Instrument, England). The degree of hemolysis in each tube was expressed as a percentage, with hemolysis in distilled water (0% NaCl) considered as 100%.

Percent Hemolysis was calculated using the formula of Faulkner et al. [26]:

$$\begin{array}{l} Percent\;(\%)\;Hemolysis=\;Optical\;density\;of\;test\;solution\;x\;100\\ \;\mathrm{Optical}\;\mathrm{density}\;\mathrm{of}\;\mathrm{standard}\;\mathrm{solution} \end{array}$$

### Statistical analyses

The data were presented as mean ± standard error of the mean (mean ± SEM). Statistical analysis was conducted using one-way analysis of variance (ANOVA) followed by Tukey post hoc test for multiple comparisons. Seasonal differences in erythrocyte osmotic fragility and vital parameters were assessed using the independent t-test. Analysis was performed using GraphPad Prism 5.0 for Windows (GraphPad Software, San Diego, CA, USA). Statistical significance was defined as P ≤ 0.05.

### Ethical statement

This study was conducted at the National Animal Production Research Institute (NAPRI) in Shika, Kaduna state (A research Center attached to Ahmadu Bello University, Zaria), after the study approval was received from the Ahmadu Bello University Committee on Animal Use and Care (ABUCAUC) with approval number of ABUCAUC/2020/56. All sample collected and data analysis was carried out at the Department of Veterinary Physiology, Faculty of Veterinary Medicine of Ahmadu Bello University, Zaria. All ethical protocols concerning the handling, care, and welfare of the animals were observed throughout the study, from its inception to its conclusion. A total of twelve donkeys were obtained from the Equine unit of NAPRI strictly for the purpose of the study and they were housed in the Beef experimental unit within the institute premises throughout the length of the study. The animal underwent screening for both endo- and ecto-parasites, confirming their apparent health status. Throughout the study period, the donkeys were provided with hay forage through zero grazing methods and were granted unrestricted access to feed and water without any alteration to the weather.

## Results

The atmospheric conditions enveloping the donkeys’ habitat during both the scorching hot-dry and rainy seasons are vividly depicted in [Table 1]. Notably, the dry bulb temperature (DBT) tended to soar during the hot-dry season, contrasting sharply with the relatively cooler temperatures of the rainy season. At the stroke of midnight during the rainy season, a minimum DBT of 12°C was registered, whereas the zenith of 25.5°C was reached at 18:00 h amid the blistering heat of the hot-dry season. In contrast, the relative humidity (RH) surged during the rainy season, peaking above 90%, while during the hot-dry season, it oscillated between 32% and 76%, markedly lower. Throughout the observation period, the temperature-humidity index (THI) remained subdued, with a nadir of 51.17 recorded at 06:00 h and a zenith of 72.14 observed at 18:00 h amidst the searing heat of the hot-dry season.

**Table 1:** Metrological condition within the stable of the donkeys during the hot-dry and rainy seasons.

Time of the day	Seasons	DBT (°C)	Relative humidity (%)	Temperature Humidity Index
00:00 h	Hot-dry	17	34	52.88
	Rainy	12	94	52.52
06:00 h	Hot-dry	16	32	51.17
	Rainy	16	95	59.36
12:00 h	Hot-dry	23.5	59	66.34
	Rainy	19	90	65.66
18:00 h	Hot-dry	25.5	76	72.10
	Rainy	24	91	71.87

**Fig 3:**
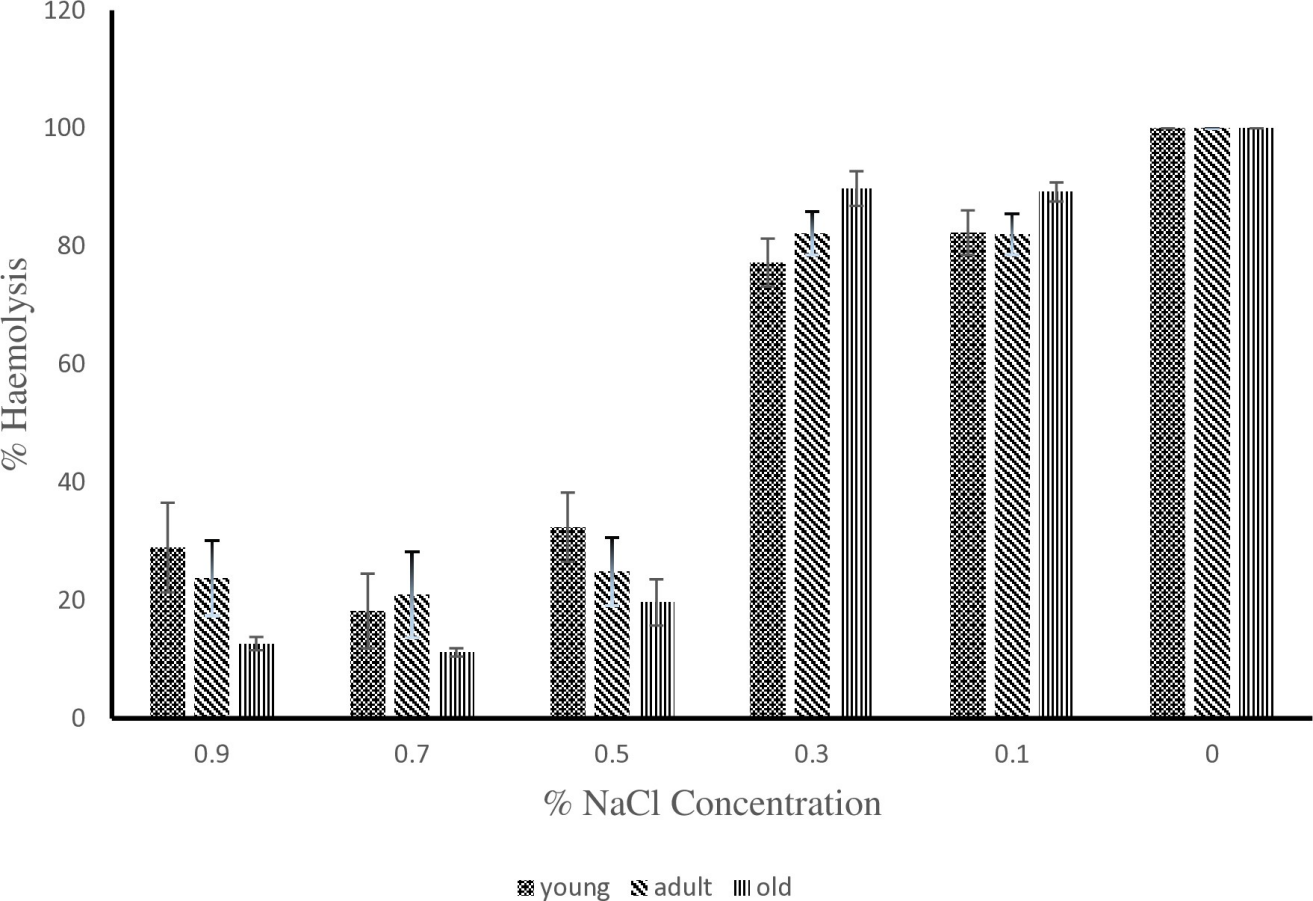
Effect of age on erythrocyte osmotic fragility of donkeys during the rainy season. ^a, b^: Means belonging to different age group and having different superscript letters are statistically significant (P < 0.05).

**Fig 4:**
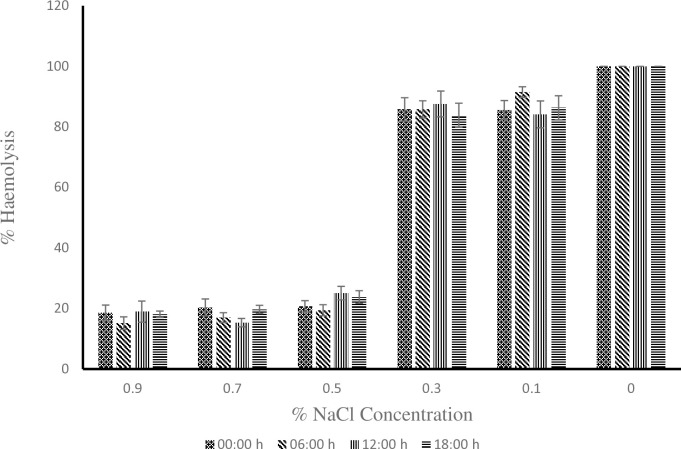
Effect of circadian rhythm on erythrocyte osmotic fragility of donkeys during the hot-dry season.

**Fig 5:**
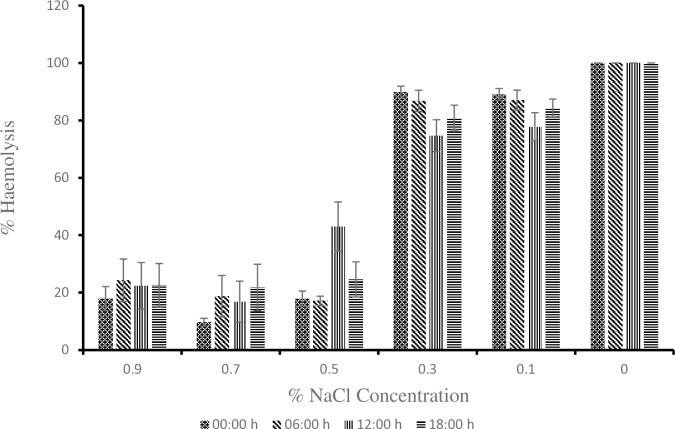
Effect of circadian rhythm on erythrocyte osmotic fragility of donkeys during the rainy season. ^a, b^: Means belonging to different hour and having different superscript letters are statistically significant (P < 0.05).

**Fig 6:**
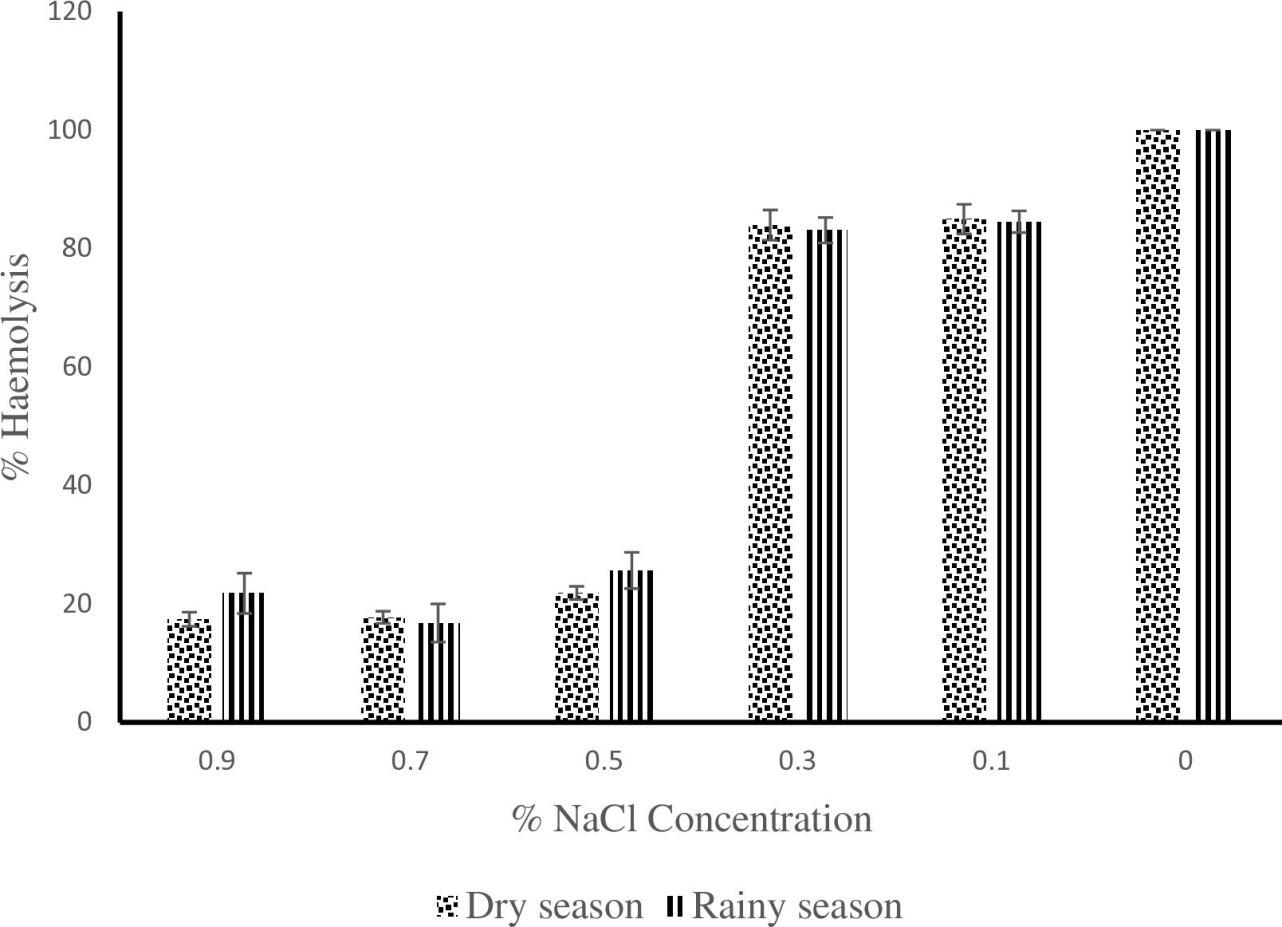
Comparison of the erythrocyte osmotic fragility of all the donkeys between seasons.

As shown in [Fig 2], at 0.3% NaCl concentration the young donkeys had the highest value of 91.04 ± 1.85 during the hot-dry season compare to the adult and old donkeys with values of 79.13 ± 3.54 and 87.12 ± 3.39 respectively. The value of 79.13 ± 3.54 obtained for the adult donkeys was statistically lower (P < 0.05) than 91.04 ± 1.85 obtained for the young donkeys. At 0.9, 0.5, 0.1 and 0% NaCl concentration, the erythrocyte osmotic fragility of young, adult and old donkeys showed no difference statistically (P > 0.05). During the rainy season as shown in [Fig 3], the old donkeys had erythrocyte osmotic fragility of 89.8 ± 2.94 which was statistically higher (P < 0.05) than the lowest value of 77.25 ± 4.07 obtained by the young donkeys at 0.3% NaCl concentration but the adult donkeys did differ from either of them (P > 0.05). At 0.9, 0.5, 0.1 and 0% NaCl concentration, the erythrocyte osmotic fragility of young, adult and old donkeys showed no statistical difference. The values for individual concentrations of NaCl for 00:00h, 06:00h, 12:00h and 18:00h timeframe from [Fig 4] showed no statistical difference from one another during the hot-dry season. From [Fig 5] at 0.5% NaCl concentration, the value of 42.98 ± 8.59 obtained at 12:00 h was statistically higher (P < 0.05) from those of 17.9 ± 2.65 and 17.15 ± 1.61 obtained at 00:00 h and 06:00 h respectively during the rainy season but the value of 24.58 ± 6.13 obtained during the 18:00 h time frame showed no statistical difference (P > 0.05). Overall, as shown in [Fig 6] the erythrocyte osmotic fragility of all the donkeys showed no statistical difference (P < 0.05) between the hot-dry and rainy seasons.

### Vital parameters and body weight of donkeys during the hot-dry and rainy season

The effect of age on rectal temperature of donkeys during the hot-dry and rainy seasons is shown in [Table 2]. During the hot-dry season, the young donkeys had the highest rectal temperature of 37.91 ± 0.09°C and the old donkeys had rectal temperature values of 37.8 ± 0.09°C which were statistically higher (P < 0.05) than rectal temperature value of 37.41 ± 0.11°C seen in the adult donkeys. More so, during the rainy season, the young donkeys also had the highest rectal temperature of 36.71 ± 0.11°C while the adult and old donkeys had 36.61 ± 0.02°C and 36.61 ± 0.21°C, respectively with no statistical significance (P > 0.05) between all the age groups. However independent t-test showed statistical difference (P < 0.05) between the rectal temperatures obtained during the hot-dry season and the rainy season in the young donkeys. The adult donkeys rectal temperature during the hot-dry season is statistically higher (P <0.05) than in the rainy season. Lastly, the rectal temperature of the old donkeys in the hot-dry and rainy season showed a level of statistical difference (P <0.05).

**Table 2:** Effect of age on vital parameters and weight of donkeys during the hot-dry and rainy season.

VITAL PARAMETERS	SEASONS	YOUNG	ADULT	OLD	TOTAL
Temperature (°C)	Hot-dry	37.91 ± 0.09^a1^	37.41 ± 0.11^b1^	37.8 ± 0.09^a1^	37.71 ± 0.06^1^
	Rainy	36.71 ± 0.11^2^	36.61 ± 0.20^2^	36.61 ± 0.21^2^	36.64 ± 0.10^2^
Respiratory rate (cycles/min)	Hot-dry	29.63 ± 0.82^1^	31.38 ± 1.48^1^	29.69 ± 1.23^1^	30.23 ± 0.69^1^
	Rainy	21.44 ± 2.45^2^	23.38 ± 2.12^2^	24 ± 2.22^2^	22.94 ± 1.29^2^
Heart rate (beat/min)	Hot-dry	48.00 ± 1.42^1^	45.56 ± 1.54	48.69 ± 1.70	47.42 ± 0.90^1^
	Rainy	55.00 ± 1.41^a2^	48.63 ± 1.53^b^	47.5 ± 1.69^b^	50.38 ± 0.99^2^
Body weight	Hot-dry	87.30± 0.77^1^	121.00 ± 0.98	139.13 ± 0.36	115.81 ± 12.40^1^
	Rainy	90.60 ± 0.68^a2^	123.70 ± 0.69^b^	140.25 ± 0.73^b^	118.18 ± 11.92^2^

^a, b^: Values along the same row with different superscript letters are statistically significant (P < 0.05), ^1, 2^: Values along the same column with different superscript numbers are statistically significant (P < 0.05).

The effect of age on the respiratory rate of donkeys during the hot-dry and rainy season is shown on [Table 2]. During the hot-dry season, the adult donkeys had respiratory rate of 31.38 ± 1.48 cycles/minute which was statistically higher (P > 0.05) than the lowest value of 29.63 ± 0.82 cycles/minute obtained in the young donkeys and 29.69 ± 1.23 cycles/minute obtained in the old donkeys. During the rainy season, the old donkeys had the highest respiratory rate of 24 ± 2.22 cycles/minute which showed no statistical difference (P > 0.05) from the lowest value of 21.44 ± 2.45 cycles/minute and 23.38 ± 2.12 cycles/minute obtained in the young and adult donkeys, respectively. Similarly, independent t-test showed that statistical significance (P < 0.05) exist between the respiratory rate of the young donkeys during the hot-dry season and the rainy season, respiratory rate of the adult donkeys during the hot–dry season and the rainy season and also statistical significance (P < 0.05) between the respiratory rate of the old donkeys during the hot-dry season and during the rainy season.

The effect of age on heart rate of donkeys during the hot-dry and rainy seasons is as shown on [Table 2]. During the hot-dry season, the old donkeys had the highest heart rate of 48.69 ± 1.70 beats/minute which showed no statistical difference (P > 0.05) from 48 ± 1.42 beats/minute and the lowest value of 45.56 ± 1.54 beats/minute recorded in the young and adult donkeys, respectively. During the rainy season, the young donkeys had the highest heart rate of 55 ± 1.41 beats/minute which showed statistical significance from the lowest value of 47.5 ± 1.69 beats/minute and 48.63 ± 1.53 recorded in the old and adult donkeys respectively. Conversely, independent t-test showed that statistical difference (P < 0.05) exist between the heart beat recorded during the hot-dry season and heart beat recorded during the rainy season in the young donkeys but no statistical difference (P >0.05) in the heart rate recorded between the two seasons in both the adult and the old donkeys.

The effect of circadian rhythm on rectal temperature is shown on [Table 3]. During the hot-dry season, the highest rectal temperature of 37.92 ± 0.07°C was recorded at 00:00h while lower rectal temperatures of 37.5 ± 0.08°C, 37.63 ± 0.18°C and 37.78 ± 0.14°C were recorded during the 06:00 h, 12:00 h, 18:00 h, respectively but no statistical difference (P > 0.05) exist between them. During the rainy season, the rectal temperature of 35.78 ± 0.17°C was recorded at 06:00 h which was statistically lower (P < 0.05) than the higher rectal temperatures of 37.11 ± 0.12°C, 37.06 ± 0.12°C and 36.62 ± 0.12°C recorded during the 18:00 h, 12:00 h and 00:00 h, respectively. The independent t-test showed that there is statistical difference (P <0.05) between the rectal temperature recorded for the donkeys during the hot-dry season and rainy season at each hour of the day.

**Table 3:** Comparison of vital parameters of the donkeys between seasons.

Vital Parameters	Dry season	Rainy season
Temperature (°C)	37.71 ± 0.06^1^	36.64 ± 0.10^2^
Respiratory rate (cycles/minute)	30.23 ± 0.69^1^	22.94 ± 1.29^2^
Heart rate (beats/minute)	50.38 ± 0.99^1^	47.42 ± 0.90^2^

^1, 2^: Values along the same row with different superscript numbers are statistically significant (P < 0.05).

The effect of circadian rhythm on respiratory rate during the hot-dry and rainy seasons is as shown on [Table 2]. During the hot-dry season, the respiratory rate of 25.5 ± 0.60 cycles/minute was recorded during the 06:00 h which was statistically lower (P <0.05) than the higher values of 33.33 ± 1.14, 30.67 ± 1.50 and 31.42 ± 1.10 cycles/minute recorded during the 00:00 h, 12:00 h and 18:00 h, respectively. During the rainy season, the respiratory rate of 36.75 ± 0.99 cycles/minute was recorded at the 12:00 h which is statistically higher (P < 0.05) than the lower values of 17.67 ± 1.42, 18.25 ± 0.75 and 19.08 ± 1.32 cycles/minute recorded at 18:00 h, 06:00 h and 00:00 h, respectively. The independent t-test showed that there was statistical difference (P <0.05) between the respiratory rate recorded for the donkeys during the hot-dry season and rainy season at each time of measurement.

The effect of circadian rhythm on heart rate of donkeys is shown on [Table 4]. During the hot dry season, the highest heart rate of 49.5 ± 1.73 beats/minute was recorded during the 00:00h which showed no statistical difference (P > 0.05) from the lower heart rates of 44.83 ± 2.35, 47 ± 1.38 and 48.33 ± 1.53 beats/minute recorded at 12:00 h, 06:00 h and 18:00 h, respectively. During the rainy season, the heart rates of 55.08 ± 1.89 beats/minute and 54.08 ± 1.83 beats/minute recorded at 18:00 h and 12:00 h respectively were statistically higher (P < 0.05) than the lower heart rates of 45.92 ± 1.42 beat/minute and 46.42 ± 1.27 beats/minute recorded at 06:00 h and 00:00 h, respectively. Also, independent t-test showed that statistical difference (P < 0.05) exist between the heart beat recorded during the hot-dry season and heart beat recorded in the donkeys during the rainy season at 12:00 h and 18:00 h but no statistical difference (P >0.05) in the heart rate recorded between the two seasons at 00:00 h and 06:00 h. The weight of young donkeys during the hot-dry season is 87.30 ± 0.77 kg, while adult donkeys weigh 121.00 ± 0.98 kg, and old donkeys weigh 139.13 ± 0.36 kg. In the rainy season, the weight of young donkeys is 90.60 ± 0.68 kg, adult donkeys weigh 123.70 ± 0.69 kg, and old donkeys weigh 140.25 ± 0.73 kg.

**Table 4:** Effect of circadian rhythm on vital parameters of donkeys during the hot-dry and rainy season.

VITAL PARAMETERS	SEASONS	00:00 h	06:00 h	12:00 h	18:00 h	TOTAL
Temperature (°C)	Hot-dry	37.92 ± 0.07^1^	37.5 ± 0.08^1^	37.63 ± 0.18^1^	37.78 ± 0.14^1^	37.71 ± 0.06^1^
	Rainy	36.62 ± 0.12^b2^	35.78 ± 0.17^a2^	37.06 ± 0.12^b2^	37.11 ± 0.12^b2^	36.64 ± 0.10^2^
Respiratory rate (cycles/min)	Hot-dry	33.33 ± 1.14^a1^	25.5 ± 0.60^b1^	30.67 ± 1.50^a1^	31.42 ± 1.10^a1^	30.23 ± 0.69^1^
	Rainy	19.08 ± 1.32^b2^	18.25 ± 0.75^b2^	36.75 ± 0.99^a2^	17.67 ± 1.42^b2^	22.94 ± 1.29^2^
Heart rate (beats/min)	Hot-dry	49.5 ± 1.73	47.00 ± 1.38	44.83 ± 2.35^1^	48.33 ± 1.53^1^	47.42 ± 0.90^1^
	Rainy	46.42 ± 1.27^b^	45.92 ± 1.42^b^	54.08 ± 1.83^a2^	55.08 ± 1.89^a2^	50.38 ± 0.99^2^

^a, b^: Values along the same row with different superscript letters are statistically significant (P < 0.05), ^1, 2^: Values along the same column with different superscript numbers are statistically significant (P < 0.05).

Lastly, independent t-test showed that the overall mean rectal temperature of 37.71 ± 0.06°C recorded during the dry season was statistically higher (P < 0.05) than 36.64 ± 0.10°C recorded during the rainy season. The overall mean respiratory rate of 30.23 ± 0.69 cycles/minute recorded during the hot-dry season in the donkeys was statistically higher (P < 0.05) than the overall mean respiratory rate of 22.94 ± 1.29 cycles/minute recorded during the rainy season. Similarly, the overall mean heart rate of 50.38 ± 0.99beats/minute recorded during the hot-dry season was statistically higher (P < 0.05) than the overall mean heart rate of 47.42 ± 0.90beats/minutes recorded during the rainy season.

## Discussion

Circadian rhythms, far from being mere responses to light and darkness, persist even in environments devoid of periodic cues, such as constant darkness [27]. Within the neural networks of the brain’s suprachiasmatic nuclei, pacemaker cells orchestrate this rhythmic symphony, regulating nerve cell firing to synchronize the circadian rhythm in donkeys [28]. This internal clock possesses an "input" mechanism, allowing for adjustment by environmental stimuli, and an "output" mechanism, governing a myriad of physiological and behavioral processes. Originally thought to be solely influenced by muscular activity and digestive processes [29], the circadian rhythm of core body temperature, as indicated by rectal temperature, is now understood to be a dynamic interplay of heat production and heat loss [30, 31]. At rest, the lion’s share of heat production stems from metabolic activities within internal organs, such as the liver, intestine, kidney, heart, and brain, collectively accounting for approximately 70% of the resting metabolic rate [31]. Meanwhile, heat loss is contingent upon environmental factors like temperature, air movement, humidity, and microclimate within stables [32].

Innovative studies, such as those by Bucklin et al. (2009) [33], have explored methods to optimize environmental conditions for animals. Their experiments with ceiling fans and sprinklers in barns demonstrated significant improvements in animal comfort compared to outdoor conditions. Sprinklers, a tried-and-tested method, induce evaporative cooling by wetting the animal’s hair coat and skin, while fans facilitate air movement for enhanced cooling efficiency [34]. Early investigations revealed that cows exhibited rapid normalization of body temperature and respiration rates when subjected to spray cooling and gentle breezes after prolonged sun exposure [35]. Further research evaluated the efficacy of spray cooling alone, demonstrating comparable or even lower rectal temperatures and respiration rates in sprinkled cows compared to shaded ones [36–38]. For horses, providing cool drinking water serves not only to replenish lost fluids but also aids in conductive heat loss, crucial for maintaining optimal body temperature [39]. Adequate monitoring ensures that equines maintain proper hydration levels, crucial for safeguarding their well-being across diverse environmental conditions. During the hot dry season, the peak dry bulb temperature (DBT) recorded at 18:00 h fell comfortably within the thermoneutral zone (22-32oC) established for donkeys in tropical regions [40]. Surprisingly, our study revealed lower DBT and relative humidity (RH) levels during this season, indicating thermal conditions conducive for the donkeys. This contrasts with prior research by Ayo et al. (1996) [41], which suggested consistently high DBT and low to medium RH during the hot dry season in northern Nigeria. The Temperature-Humidity Index (THI) serves as a gauge for thermal stress in animals [42]. Despite reaching a peak THI of 72.10 at 18:00 h, falling below the threshold of 80 [43], indicative of thermal comfort, this could be attributed to the ample shade provided by numerous trees in the study area, along with favorable wind speeds. Similarly, during the rainy season, the peak DBT also fell within the thermoneutral zone for tropical donkeys [40]. However, the high THI of 71.87 recorded at 18:00 h was below the comfort threshold of 80 established for donkeys, signaling potential discomfort. Furthermore, the elevated RH observed at 06:00 h during the rainy season exceeded the thermoneutral zone of 30–70% [44], consistent with Ayo et al.’s (1996) findings [41] of medium-level DBT and very high RH during this season. These environmental nuances underscore the importance of understanding local climate dynamics in managing equine welfare effectively.

The research findings vividly illustrate that during the hot-dry season, young and old donkeys exhibited heightened erythrocyte hemolysis compared to their adult counterparts. This suggests that the erythrocyte membranes of adult donkeys were notably more resilient, capable of withstanding considerable stress in contrast to the fragility observed in the young and old donkeys. Such differences likely stem from the juvenility of young donkeys, where erythrocytes are inherently more fragile compared to adults. This aligns with prior research by Oyewale et al. (1998) [44], which observed increased fragility in erythrocytes of younger ducks due to variations in cholesterol-phospholipid ratios within the membrane. Interestingly, the high erythrocyte hemolysis in old donkeys contradicts findings by Basarab *et al*. (1980) [45], suggesting decreased osmotic fragility with age in cattle. Similarly, during the rainy season, elevated % hemolysis in old donkeys deviates from Mosior et al.’s (1985) [46] observations of decreased fragility in older human and bovine erythrocytes. This discrepancy may reflect waning immunity and stress tolerance in aging donkeys. Additionally, the study unveils a novel insight: erythrocytes at 12:00 h exhibited greater fragility compared to other times of the day, potentially due to increased afternoon sunshine during the rainy season, despite shaded stable conditions.

Contrary to expectations, comparisons across seasons revealed no statistically significant differences in erythrocyte osmotic fragility, contradicting reports of increased fragility in Red Sokoto and Sahel goat kids during the hot-dry season [47]. Moreover, during the hot-dry season, young donkeys exhibited higher mean rectal temperatures than adults and old donkeys, a trend consistent during the rainy season as well. This aligns with findings by French et al. (1995) [48], suggesting that the high metabolic rates of young donkeys contribute to elevated temperatures. Interestingly, the highest rectal temperature during the rainy season occurred at 18:00 h, akin to findings by Canacoo *et al*. (1991) [49] of a noon temperature of 37.8 ± 0.2 oC. Overall, mean rectal temperatures were higher during the hot-dry season, likely due to increased heat dissipation to the environment during this period.

The respiratory rate exhibited no statistically significant differences between the hot-dry and rainy seasons. However, noteworthy patterns emerged: adult donkeys showed higher respiratory rates during the hot-dry season, whereas old donkeys exhibited elevated rates during the rainy season, diverging from findings by French et al. (1995) [48], which observed high rates in young donkeys. Interestingly, the highest mean rectal temperature occurred at 00:00 h during the hot-dry season and at 12:00 h during the rainy season, aligning with French et al.’s (1995) [48] observations. All recorded values fell within the normal respiratory rate range, indicating that the donkeys remained within the tolerance index. As anticipated, respiratory rates during the hot-dry season surpassed those of the rainy season, likely serving as a physiological mechanism for heat dissipation in hot conditions.

Though not statistically different, heart rate values exceeded the normal range (38–45 beats/minute) for tropical donkeys during the hot-dry season [40]. Young donkeys exhibited particularly high heart rates, potentially attributable to their higher metabolic rates and increased body surface area to volume ratio [50]. This trend aligns with Ohmura et al.’s (2017) [51] findings, demonstrating age-related decreases in heart rate from foals to old horses in thoroughbred breeds. Elevated heart rates across all donkeys during the hot-dry season may signify heightened cardiac output to dissipate heat. Moreover, the increase in heart rate across seasons suggests an effort by the animals to enhance heat loss, possibly via radiation, conduction, and convection from the skin surface to the atmosphere. The highest heart rate recorded at 18:00 h during the rainy season corroborates findings by [11], which noted peak values between 18:00 h and 20:00 h in horses. The variation in the average weight of donkeys across different age groups and seasons might indicate the influence of environmental factors, nutrition, and metabolic changes on the animals’ growth and body condition. During the hot-dry season, different donkey age groups have different weight. These values are slightly lower compared to the rainy season. The seasonal differences can be attributed to the availability of lush forage and water resources during the rainy season, which likely supports better nutrition and weight gain [52]. Previous study by Ayo et. al. [20] have also shown that donkeys, like other livestock, tend to gain more weight during the wet season when food and water are more plentiful, leading to better body conditions and overall health [53]. Similarly, the decrease in body weight observed during the hot-dry season can be linked to heat stress and reduced feed quality, which negatively impacts feed intake and metabolic efficiency. Heat stress is known to induce physiological changes in donkeys, including a reduction in feed intake and altered water balance.

## Conclusion

The recorded meteorological parameters shed light on the thermal comfort experienced by the donkeys across seasons. During the hot-dry season, the combination of low dry bulb temperature (DBT) and relative humidity indicated favorable thermal conditions for the animals. Moreover, the thermal comfort index (THI) remained below the threshold of 80, affirming a state of comfort even at its peak at 18:00 h. Similarly, in the rainy season, the DBT remained within the thermoneutral zone for tropical donkeys, suggesting adequate environmental conditions. However, beyond the climatic changes, the study unearthed some insights into the physiological responses of the donkeys to seasonal variations. Particularly during the hot-dry season, both young and old donkeys exhibited heightened erythrocyte hemolysis compared to adult counterparts. Conversely, in the rainy season, old donkeys experienced elevated hemolysis levels, indicating potential age-related differences in stress tolerance. Furthermore, the physiological parameters of rectal temperature and heart rate revealed notable patterns. Regardless of the season, young donkeys consistently displayed the highest rectal temperatures and heart rates, suggestive of their heightened metabolic activity. Interestingly, across seasons, the hot-dry period emerged as the time when donkeys experienced peak physiological responses, evident in higher rectal temperatures, respiratory rates, and heart rates. While climatic conditions play a crucial role in shaping the thermal environment for donkeys, their physiological responses to seasonal variations unveil a great interplay between environmental cues and internal mechanisms. These findings revealed the balance between adaptation and vulnerability in equine (especially donkey) physiology, highlighting the need for comprehensive management strategies tailored to seasonal dynamics.

## Recommendation and limitations of the study

To foster the well-being of donkeys, it is recommended to continue using the housing strategy employed in this study, as it demonstrated an ability to mitigate stress levels effectively. A more comprehensive approach to management practices should also be implemented, particularly when caring for young and elderly donkeys, which may require tailored attention to cope with environmental stressors. Given the findings related to age-related differences in stress tolerance, adult donkeys should be prioritized for more physically demanding activities, such as packing, as they exhibit greater resilience compared to their younger or older counterparts.

To deepen our understanding of donkey physiology, future research should include a gender analysis, exploring potential differences in stress and metabolic responses between male and female donkeys. Additionally, expanding the study to other seasons and geographic regions would provide a broader perspective on how different environmental conditions impact donkey welfare. Including a wider range of physiological markers (e.g., cortisol levels, immune responses) and behavioral assessments would allow for a more comprehensive understanding of how donkeys respond to environmental changes.

Finally, future studies should consider the role of nutrition and hydration, given their critical impact on thermoregulation and overall health, and should incorporate longitudinal data to observe the long-term effects of seasonal and environmental variations on donkey well-being. By addressing these limitations and recommendations, researchers and animal caretakers can contribute to the creation of environments that promote the health, comfort, and vitality of donkeys across various climates and seasons.

## Supporting information

S1 File (XLSX)

S2 File (XLSX)

S3 File (PDF)
