# Supplementary material for: Effects of age and circadian rhythm on vital parameters and erythrocyte osmotic fragility of donkeys during seasonal changes
Source: PLoS One. 2025 Jan 31;20(1):e0313780. doi: 10.1371/journal.pone.0313780 (PMC11785283; doi:10.1371/journal.pone.0313780)
Supplement: S3 File — (PDF) [file pone.0313780.s003.pdf]

Effect of Age and Circadian Rhythm on vital Parameters of Donkeys

Aim: To evaluate age and circadian rhythm effects on vital parameters and erythrocyte osmotic fragility in donkeys.

Methods:

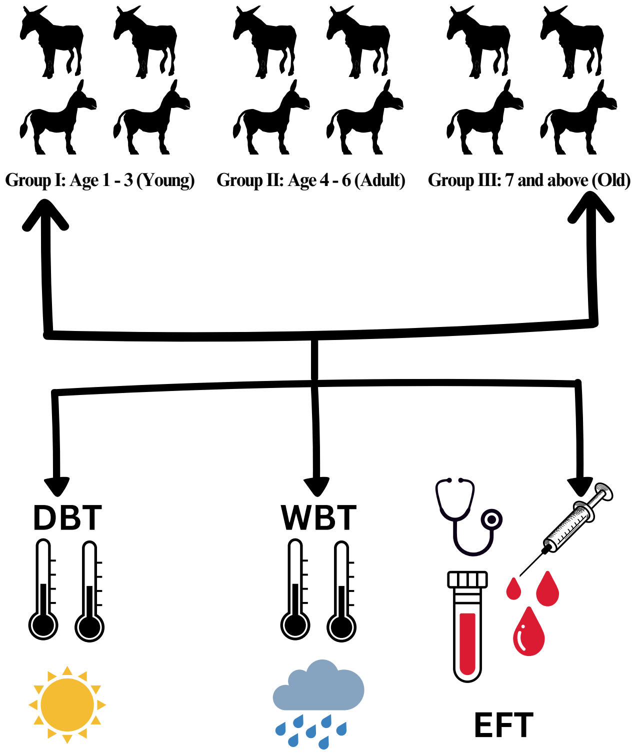

Figure 1: Three age groups (Young, Adult, Old) with four donkeys each, monitored for 24 hours during hot-dry and rainy seasons.

Results:

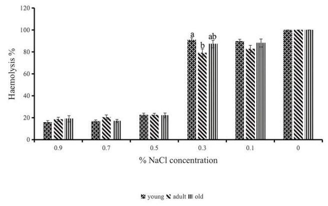

Figure 2: Effect of age on erythrocyte osmotic fragility of donkeys during the hot-dry season.
a, b: Means belonging to different age group and having different superscript letters are statistically significant (P < 0.05).

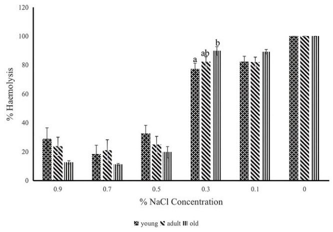

Figure 3: Effect of age on erythrocyte osmotic fragility of donkeys during the rainy season.
a, b: Means belonging to different age group and having different superscript letters are statistically significant (P < 0.05).

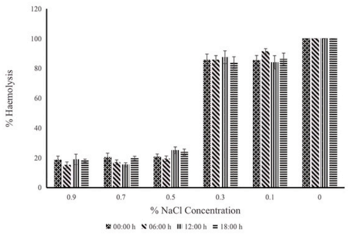

Figure 4: Effect of circadian rhythm on erythrocyte osmotic fragility of donkeys during the hot-dry season.

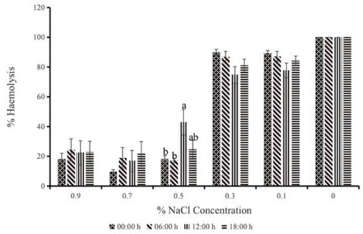

Figure 5: Effect of circadian rhythm on erythrocyte osmotic fragility of donkeys during the rainy season.
a, b: Means belonging to different hour and having different superscript letters are statistically significant (P < 0.05).

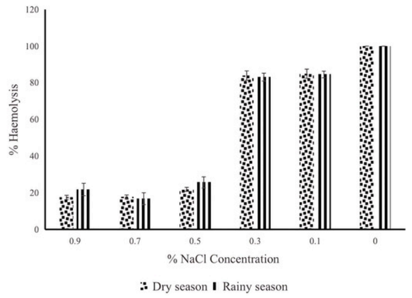

Figure 6: Comparison of the erythrocyte osmotic fragility of all the donkeys between seasons.

Discussion: During the hot-dry season, the young donkeys had mean rectal temperature of 37.91 ± 0.09 oC and the old donkeys had mean rectal temperature values of 37.8 ± 0.09 oC which were statistically higher (P < 0.05) than the mean rectal temperature value of 37.41 ± 0.11 oC seen in the adult donkeys. However during the rainy season, the heart rates of 55.08 ± 1.89 beats/minute and 54.08 ± 1.83 beats/minute recorded at 18:00 h and 12:00 h respectively were statistically higher (P < 0.05) than the lower heart rates of 45.92 ± 1.42 beat/minute and 46.42 ± 1.27 beats/minute recorded at 06:00 h and 00:00 h, respectively. While Old donkeys had higher Erythrocytes Osmotic fragility during rainy season.

Conclusion: Hot-dry season showed higher dry-bulb temperatures (DBT), while rainy season had lower DBT and higher humidity. Proper management recommended for young and old donkeys during hot-dry season.

Keywords: Erythrocytes osmotic fragility, Physiology, Seasonal changes, Circadian rhythm, Donkey.
